# The relationship between Quality and Outcomes Framework scores and socioeconomic deprivation: a longitudinal study

**DOI:** 10.3399/BJGPO.2023.0024

**Published:** 2023-12-13

**Authors:** Oliver Mann, Tristan Bracegirdle, Saran Shantikumar

**Affiliations:** 1 Warwick Medical School, University of Warwick, Coventry, UK

**Keywords:** Quality and Outcomes Framework, Low socioeconomic status, Primary health care, Health care quality assessment

## Abstract

**Background:**

The Quality Outcomes Framework (QOF) is a pay incentive scheme in England designed to improve and standardise general practice. QOF attainment has been used as a proxy for primary care quality in previous research.

**Aim:**

To investigate whether there is a relationship between socioeconomic deprivation and QOF attainment in primary care in England.

**Design & setting:**

Retrospective longitudinal study of primary care providers in England.

**Method:**

QOF scores were obtained for individual general practices in England from between 2007–2019 and linked to practice-level Indices of Multiple Deprivation (IMD) scores derived from census data. Beta regression analyses were used to analyse the relationship with either percentage of total QOF attainment or of domain-specific attainment with multivariate analyses, adjusting for additional practice-level demographics. QOF attainment in the most affluent quintile was used as the reference group.

**Results:**

General practices in less deprived areas have consistently outperformed those in more deprived areas in terms of QOF achievement. Initially, the gap between least and most deprived practices decreased, however since 2015 there has been relatively little change in comparative performance. The magnitude of inequality was reduced after adjusting for demographic factors. Of the independent variables analysed, the proportion of patients aged >65 years (‘over 65s’) had the strongest relationship with QOF attainment.

**Conclusion:**

There remains an inequality in primary care quality by socioeconomic deprivation in England, even after accounting for demographic differences.

## How this fits in

As a financial incentive, QOF scoring has the potential to influence the organisation and clinical practice of GPs. QOF scores were in part introduced to standardise and subsequently equalise primary healthcare. Initial analyses of QOF scoring after its introduction showed that it may have marginally improved GP quality. Further appraisal of its effectiveness in reducing healthcare inequality over a longer time period would be valuable, especially after its discontinuation in some countries of the UK.

## Introduction

Financial incentives have been proposed as a potential mechanism to address healthcare provision inequalities.^1^ In 2004, the UK government introduced a pay-for-performance element to the new general practice contract called the ‘Quality and Outcomes Framework’ (QOF), with an eye towards reducing health inequality.^2,3^ While voluntary, almost all general practices in England participate and it has become a valuable source of information. QOF measures whether a general practice surgery achieves incentivised clinical and non-clinical ‘indicators’, with a score being given according to the percentage of patients for whom the indicators are met. These targets, for example whether people with atrial fibrillation are treated with anticoagulation therapy, are curated by a National Institute for Health and Care Excellence (NICE)-appointed body and are generally considered grounded in evidence-based medicine.^4^ Therefore, QOF has become regarded as a surrogate marker of general practice quality.

Despite discussion on whether QOF is a true marker of healthcare provision performance, other groups have previously investigated the relationship between QOF achievement and socioeconomic deprivation. They have shown that there was some improvement in care quality following the introduction of QOF, although the degree to which this has reduced inequality is debated.^3,5–8^

To the authors’ knowledge, no recent analyses of QOF achievement and deprivation are available in peer-reviewed sources. This study aimed to evaluate whether the relationship between socioeconomic deprivation and QOF achievement has changed over the last 15 years.

## Method

### Data collection

The authors obtained QOF achievement data for all general practices in England for 2007–2019 from NHS Digital (https://qof.digital.nhs.uk/search/). Data from 2020 were deliberately excluded from this study due to the unknown potential effects of the COVID-19 pandemic on QOF reporting completeness and accuracy. The number of practices for which data were available ranged from 6873–8372, with fewer practices in the later datasets. QOF scores between 2014 and 2019 were available in total, as well as by three specific domains (*clinical*, *public health*, and *public health additional*). For the years 2007 to 2013, only QOF total scores and QOF clinical domain scores were available. The QOF data, both in total and domain-specific, were then expressed as a percentage of maximum points available for each corresponding year, as the maximum number of points available varied each year. NHS Digital was also the source of primary care practice demographic data, including list size (number of registered patients), number of females, and number of over 65s, which was available and obtained for each practice from 2013 onwards (https://digital.nhs.uk/data-and-information/publications/statistical/patients-registered-at-a-gp-practice; *Series/Collection, Patients Registered at a GP Practice*, NHS Digital). Demographic data were available for between 7007 and 8106 practices.

This study used Index of Multiple Deprivation (IMD) scores as a marker of socioeconomic deprivation, a composite measure that accounts for underlying area-based factors that contribute to socioeconomic deprivation, including employment, income, and housing. They were first issued in 2010, with scores updated in 2015 and 2019. IMD scores pertaining to individual primary care practices were obtained from Public Health England’s *Fingertips* repository (https://fingertips.phe.org.uk/). Data were available for 6407 (IMD 2010), 6529 (IMD 2015), and 6545 (IMD 2019) practices, respectively.

QOF scores, IMD scores, and demographic data were linked using unique GP practice codes as the linkage variable, to produce a single dataset for each year of analysis. Complete information was available for between 6302 and 6949 practices, depending on the year, and practices that did not have the complete dataset were excluded. Any practice with a list size of under 1000 patients was also excluded from further analysis, to remove out-of-hours and walk-in centres which may have been included in the primary data sources. Between 89 and 135 practices were excluded, varying per year.

### Data analysis

For each year, this study analysed the association between IMD scores (independent variable) and percentage achievement for QOF total and domain-specific scores (dependent variable), using univariate beta regression. For the years 2013 onwards, where practice demographic data were available, this study also conducted multivariate beta regression to additionally adjust for 1) practice list size, 2) the proportion of female patients, and 3) the proportion of patients aged 65 years and older. Due to the non-parametric distributions of the independent variables, each was stratified into quintiles (where quintile one represented the lowest 20% of values and quintile five represented the highest 20% of values) prior to regression analysis. All analyses were conducted in the statistical software R (Version 4.1.2), using R Studio.

## Results

### Crude association between deprivation and total QOF achievement (2007–2019)

**Figure 1.**
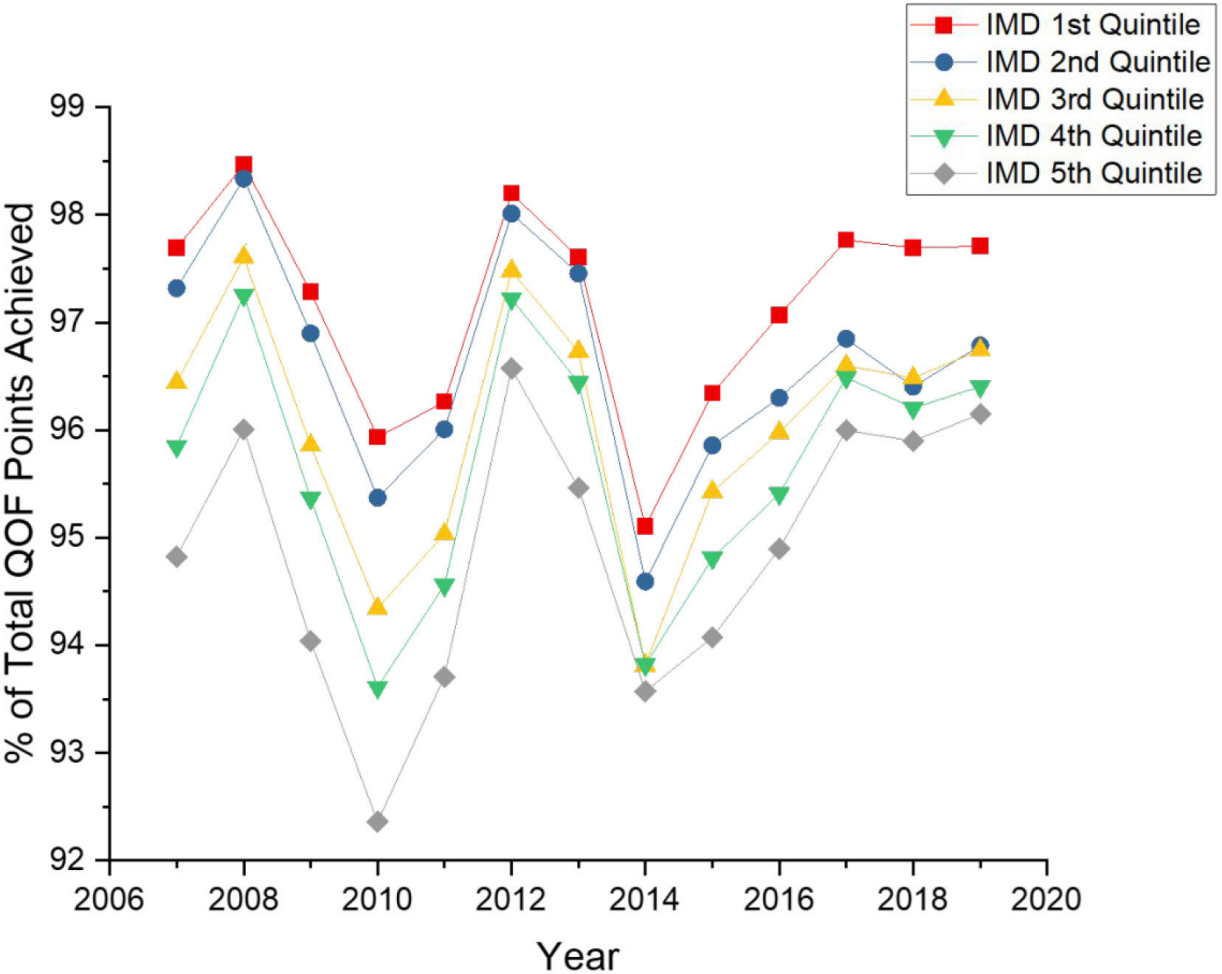
Total percentage of available QOF points achieved 2007–2019 on average for each IMD quintile IMD = Index of Multiple Deprivation. QOF = Quality Outcomes Framework.

To accommodate for changes in the QOF framework throughout time, this study tracked changes in average total QOF points achieved as a percentage of total points available for each year, stratified by practice IMD quintiles. A step-wise trend of less deprived practices having higher QOF attainment than more deprived areas was seen. This study also observed that all IMD quintiles shared similar fluctuations in the percentage of available points earned (Figure 1).

**Figure 2.**
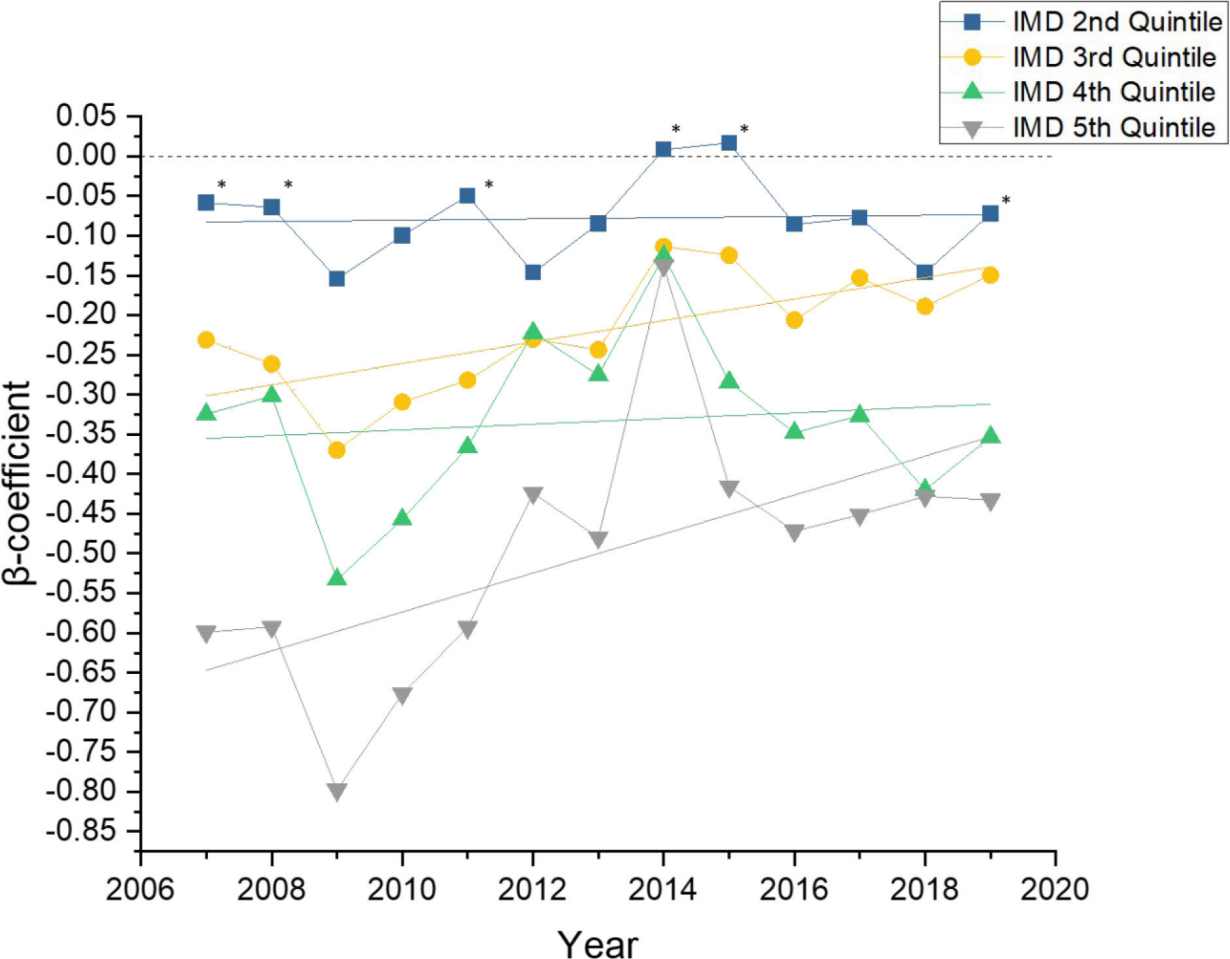
Univariate beta coefficients 2007–2019 comparing the least deprived practices (quintile 1) to quintiles 2–5 of deprivation for overall QOF achievement. Points marked with an asterisk (*) indicate values with 95% confidence intervals overlapping the quintile one value for that given year. IMD = Index of Multiple Deprivation.

Due to these shared variations, the authors further decided that comparisons to the achievement of least deprived practices (quintile 1) by univariate beta regression analysis would better aid in exploring trends between QOF achievement and deprivation. The beta coefficients of regression demonstrate the proportional difference in percentage QOF achievement for a given quintile of practices compared to the least deprived practices. This analysis, as shown in Figure 2, demonstrated that GP practices in the third to fifth quintile of deprivation had a statistically significantly lower total QOF achievement than those in the least deprived quintile, with a step-wise reduction in achievement for each quintile of increasing deprivation. Linear fit analysis was performed for each series of quintile results, with all groups tending towards the achievement of the least deprived practices (Figure 2).

### Adjusted association between deprivation and total QOF achievement (2013–2019)

**Figure 3.**
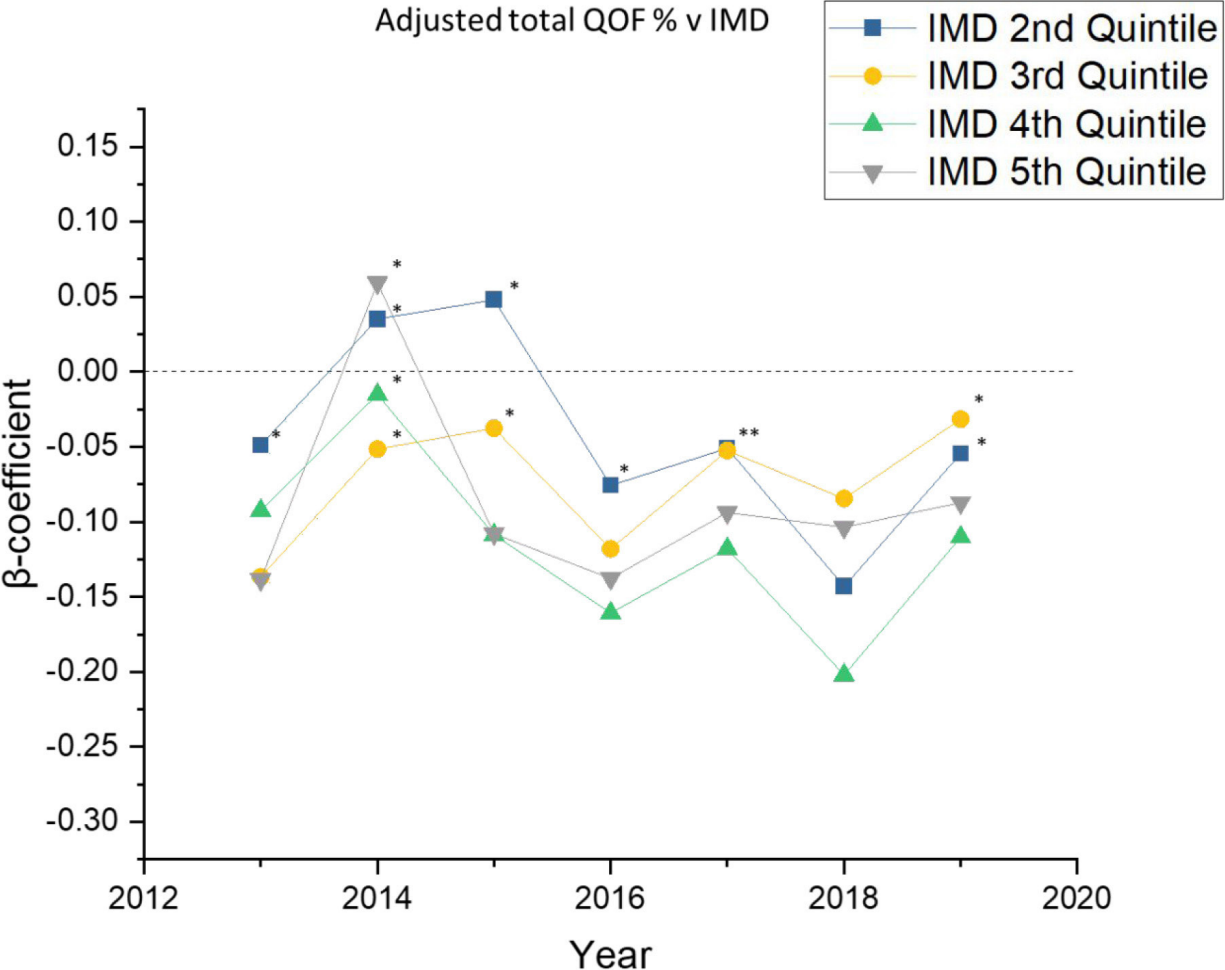
Multivariate beta coefficients 2013–2019 comparing the least deprived practices (quintile 1) to quintiles 2–5 of deprivation for overall QOF achievement. Points marked with an asterisk (*) indicate values with 95% confidence intervals overlapping the quintile one value for that given year. IMD = Index of Multiple Deprivation. QOF = Quality Outcomes Framework.

To increase the confidence that differences in QOF attainment between deprivation quintiles were not due to demographic differences in patient populations, analyses were adjusted to account for differences in the size of GP practices, the female-to-male ratio, and the proportion of over 65s (where data were available). Doing so complicated interpretation of the data, with the previously seen step-wise relationship no longer clearly apparent (Figure 3). Importantly, the magnitude of the beta coefficient values compacted closer together and closer to zero. Although these changes suggested that some of the differences in QOF achievement can be attributed to practice demographics, the primary care practices in the most deprived areas almost always returned worse QOF performances compared to the least deprived practices.

### Total QOF achievement by practice demographics

**Figure 4.**
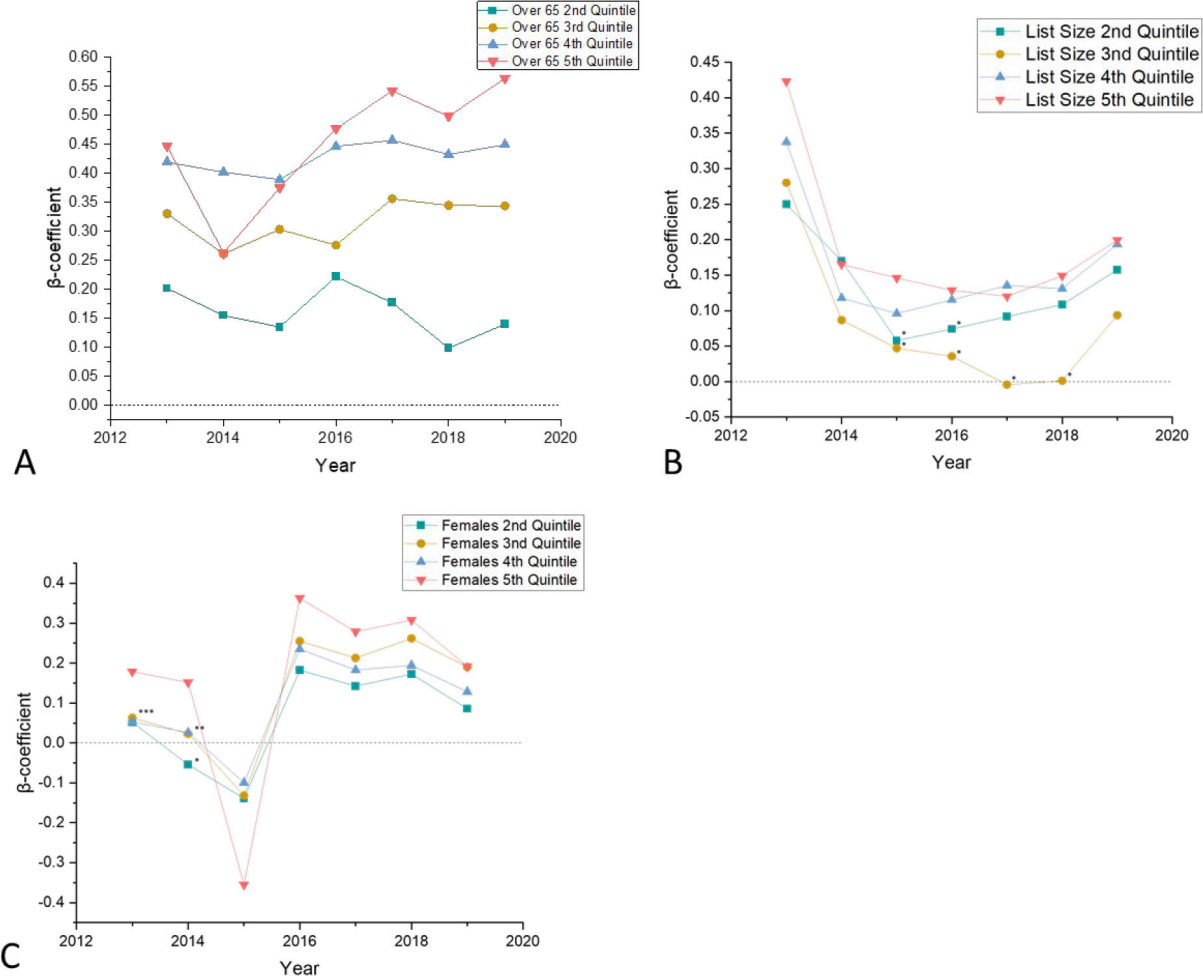
Comparisons of total Quality Outcomes Framework (QOF) achievement between quintile 1 and quintiles 2–5 in different general practice characteristics, 2013–19.

Seeing the difference adjusting for demographic factors had on QOF achievement, the authors decided to investigate their individual impact. One at a time, this study grouped these variables into quintiles as before, and adjusted QOF performance for IMD and the remaining two variables with regression analyses. The strongest link was that a higher proportion of over 65s lead to consistently higher QOF achievement (Figure 4A), with a reverse step-wise relationship in most years. The impact of GP list size (Figure 4B) and sex ratio (Figure 4C) was more inconsistent, with the erratic and sometimes statistical insignificance of some data points meaning caution must be used in drawing conclusions.

Figure 4A compares QOF scores between practices with the fewest patients over 65 (quintile 1) to practices with a larger proportion of over 65s (quintiles 2–5) . Figure 4B compares QOF scores between practices with the smallest list size (quintile 1) to practices with larger list sizes (quintiles 2–5). Figure 4C compares QOF scores between practices with the smallest percentage of females (quintile 1) to practices with a larger percentage of females (quintiles 2–5).

### Domain-specific QOF achievement by deprivation

**Figure 5.**
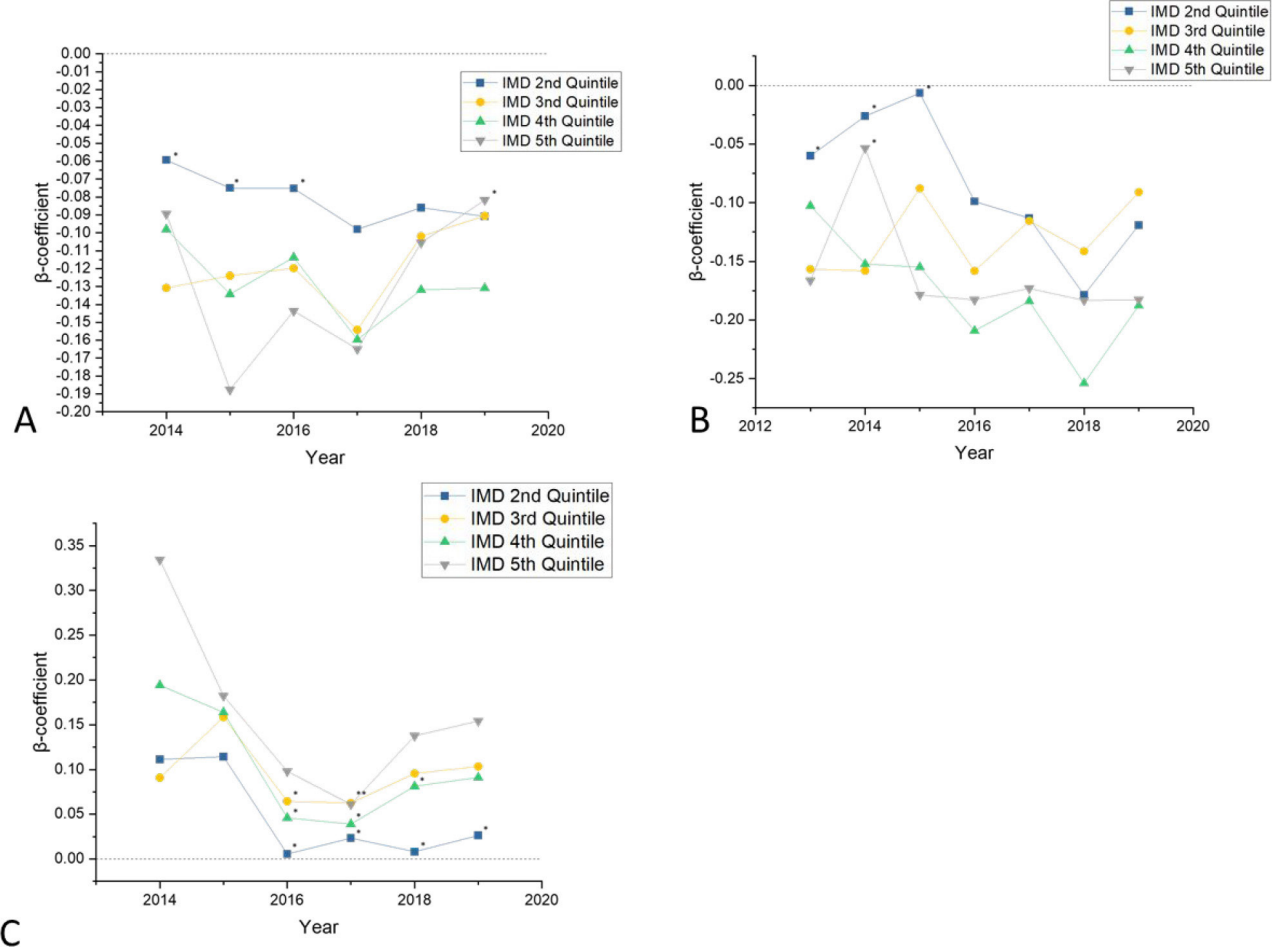
Multivariate analysis results of QOF achievement between quintile 1 to 2–5 in each domain of QOF 2013–19. IMD = Index of Multiple Deprivation. QOF = Quality Outcomes Framework.

To this point, only total QOF achievement has been discussed. However, QOF points are presently assigned into one of three domains: clinical, public health, and public health additional. To uncover any relationship between deprivation and particular types of QOF outcomes, this study compared individual domain performance to IMD quintiles, adjusted for the demographics previously discussed. For the clinical domain, this study detected that GP practices in more deprived areas appeared to achieve fewer clinical domain QOF points (Figure 5A). A similar pattern was elucidated for public health additional domain QOF points (Figure 5B), with more deprived areas generally achieving fewer QOF points in this domain. However, the public health domain showed a reverse in this trend, with more deprived practices achieving more public health QOF points (Figure 5C).

Figure 5a assesses the clinical domain, Figure 5b assesses the public health additional domain, and Figure 5c assesses the public health domain.

## Discussion

### Summary

The authors performed a decade-spanning longitudinal study of QOF achievement for GP practices in England with the objective of determining the relationship between QOF attainment and deprivation, as defined by IMD score. This study found mixed results that make defining that relationship with certainty difficult.

Using unadjusted analyses for total QOF achievement, GP practices in the least deprived areas consistently outperformed those in the most deprived areas between 2007–2019. However, the overarching trend of this study was an incremental progress towards equality, until 2012, where the data saw a levelling off. After adjusting for practice demographics, this study found a narrower gap between the most and least deprived general practices throughout the years 2013–19, when data were available, but no clear trend of improvement or increasing inequality. These results suggested that some of the inequality in QOF outcome performance may be due to underlying practice-level factors not related to deprivation, although the potential reasons for this cannot be elucidated from the present analysis.

The authors concluded from this study’s analyses of outcomes for specific QOF domains that more deprived practices had fewer clinical and public health additional domain points, contrasted to higher public health domain QOF scores. The authors speculate that the reason for this association is the strong link between prevalence of chronic disease and the ability to achieve QOF points, particularly in the public health domain, and it is known that prevalence of chronic disease is higher in more deprived areas.^9,10^

The public health additional domain is of interest, as it only currently assesses two indicators of cervical screening. Lower uptake of cervical screening in the most socioeconomically deprived areas has been reported.^11,12^ The results from this study extend on these findings, showing that second and third deprivation quintiles also achieved lower cervical screening QOF scores than the least deprived areas.

This study’s demographic analyses showed much higher QOF scores in practices with a higher proportion of over 65s. The authors similarly propose chronic disease prevalence as an explanation for this, due to the nature of the QOF scoring system. If disease prevalence truly is improving QOF attainment, it may be expected that more deprived areas, which typically have higher disease prevalence, also have the potential for higher QOF scores.^13^ As such, QOF may be underestimating the difference in health between the most and the least deprived areas.

### Comparison with existing literature

Previous research in this area has almost exclusively focused on short periods of time without analysing longitudinal trends. Although this study comes to the same conclusion as those written by other groups,^5,9,14–16^ the authors succeeded in extending on these conclusions by reviewing a longer timeframe.

In line with Crawley *et al*, the authors suggest that QOF has had little significant impact on healthcare inequality in England over an extended period.^8^ However, it remains uncertain whether there have been any absolute improvements in primary care quality due to QOF implementation.

### Strengths and limitations

The ability of QOF to accurately reflect healthcare provision and its impact on health inequalities has been widely discussed elsewhere.^4,5,7,17–19^ Exception reporting, a mechanism to ensure practices were not penalised for factors such as missed appointments and contraindications for recommended drug prescription, has the possibility to skew this study’s findings, given that exception reporting may be disproportionate across the deprivation gradient.^20^ Further, QOF has gone through many refinements over time with greater change in some years rather than others, limiting the conclusions that can be drawn about QOF trends over time.

It was not possible to obtain for this study publicly available information on QOF scores from before 2007 and key demographics used in adjustments from before 2013. Noting the previously reported effect that GP density, training status, and single-handed practices have on QOF attainment,^9,14^ the authors would ideally have also analysed these variables.

This study used IMD data to assess deprivation. A single IMD score was attributed to each practice, and therefore may not reflect the range of socioeconomic status of all the patients within that practice.^9^

### Implications for research and practice

Further research is needed to confirm this study’s findings, including through adjustment of other practice-level factors like chronic disease prevalence, as well as to understand the underlying drivers of inequality such that interventions can be developed and funded to address them.

It is possible to see why QOF may contribute to healthcare inequalities as better performing practices receive greater financial support, possibly multiplying inequalities over time. Despite this, less than 10% of GP funding comes from QOF, and the absolute difference between the most and the least deprived quintiles is only around 3%.^21^ Therefore, it is unlikely that QOF *contributes* to inequality. It would be interesting to see if the QOF incentive scheme has had a lasting impact on the average healthcare standard in primary care focusing on outcomes both related and unrelated to QOF.

Gillam and colleagues commented that the introduction of QOF brought about small improvement to the gaps between most and least deprived QOF achievement.^18^ Extending the evaluation for the subsequent decade found that inequalities in quality have not significantly improved, so more work is required to reduce the disparity between the most deprived areas and the least deprived areas observed in this study.

## Data

The datasets relied on in this article can be sourced from the corresponding author on reasonable request, should they no longer be accessible on the public domains cited.
